# Human transgenerational observations of regular smoking before puberty on fat mass in grandchildren and great-grandchildren

**DOI:** 10.1038/s41598-021-04504-0

**Published:** 2022-01-21

**Authors:** Jean Golding, Steve Gregory, Kate Northstone, Marcus Pembrey, Sarah Watkins, Yasmin Iles-Caven, Matthew Suderman

**Affiliations:** Population Health Sciences, Bristol University, Bristol, UK

**Keywords:** Epidemiology, Outcomes research

## Abstract

Previously, using data from the Avon Longitudinal Study of Parents and Children (ALSPAC) we showed that sons of *fathers* who had started smoking regularly before puberty (< 13 years) had increased fat mass during childhood, adolescence, and early adulthood. We now show that if the *paternal* grandfather had started smoking pre-puberty, compared with later in childhood (13–16 years), his granddaughters, but not grandsons, had evidence of excess fat mass at two ages: mean difference + 3.54 kg; (P with 1-tailed test) = 0.043 at 17 years, and + 5.49 kg; (P_1_ = 0.016) at age 24. When fathers of *maternal* grandfathers had started smoking pre-puberty, their great-granddaughters, but not great-grandsons, had excess body fat: + 5.35 kg (P_1_ = 0.050) at 17, and + 6.10 kg (P_1_ = 0.053) at 24 years. Similar associations were not found with lean mass, in a sensitivity analysis. To determine whether these results were due to the later generations starting to smoke pre-puberty, further analyses omitted those in subsequent generations who had smoked regularly from < 13 years. The results were similar. If these associations are confirmed in another dataset or using biomarkers, this will be one of the first human demonstrations of transgenerational effects of an environmental exposure across four generations.

## Introduction

Recent studies concerning associations between ancestral exposures and health effects on their successors have been prompted by a detailed comparison of the survival of individuals born on the edge of the Arctic Circle between 1880 and 1915 in the village of Ӧverkalix. Their grandparents’ exposures to famine and/or a harvest glut during their own childhoods was identified and details of their ages at the time linked to their grandchildren’s health indices. Analyses highlighted the following: (a) there were strong relationships which were sex-specific, both regarding the sex of the exposed grandparent and the sex of the affected grandchild, and (b) the exposure effects were specific to particular ages of exposure—the most susceptible period being pre-puberty, defined as ages 8–10 years for girls and 9–12 years for boys^1^.

A subsequent project by Van den Berg and Pinger^2^ studied the children and grandchildren of individuals who were exposed to the Berlin famine. They demonstrated that the grandsons whose *paternal* grandfathers had been exposed to the famine prepuberty (age 8–12 years) had higher (better) mental health scores. In parallel granddaughters had higher mental health scores if their *maternal* grandmothers had experienced the famine during the pre-puberty ages.

These findings, together with information from experimental studies, has prompted Soubry after reviewing the literature, to stress the need to explore paternal contributions to the offspring’s health. She went on to suggest that just as there is evidence supporting the concept of the Developmental Origins of Health and Disease (DOHaD), there is increasing evidence for paternal influences, which she coined the Paternal Origins of Health and Disease (POHaD)^3^. The most recent population studies to investigate this have largely focussed on asthma as the outcome of interest. The authors have shown paternal exposures to smoking of cigarettes prepuberty to be associated with increased risk of asthma and reduced lung function as well as of increased fat mass in the offspring^4^.

The number of studies that have been able to look at paternal influences on an outcome is small, but nonetheless compelling, especially animal studies which have led some to hypothesise about mechanisms, e.g.^5–7^. Still smaller are studies examining associations with the outcome of the grandchild, apart from the Overkalix studies. Assessment of outcomes of great-grandchildren is almost certainly unique. Information on environmental exposures during the childhood of parents has been collected only occasionally, and that of grandparents rarely among major population birth cohort studies. The Avon Longitudinal Study of Parents and Children (ALSPAC) is one pre-birth cohort which has collected information on the ages at which the parents of the study children had started smoking regularly. These data have been used to determine whether a parent starting smoking prepuberty was associated with the weight of their offspring. We demonstrated that if the fathers had started smoking regularly prior to 11 years of age, their sons (but not their daughters) were more likely to have an increased body mass index (BMI), largely associated with excess fat mass at ages 13, 15 and 17^8^. A subsequent detailed study of antecedents associated with fat mass at age 24 showed that a similar association remained with paternal onset of smoking < 11 years, which increased in size on adjustment for confounders^9^. There is support for these findings since the association of fathers’ prepubertal onset of smoking with fat mass in sons but not daughters was also found in the RHINESSA study^4^.

These findings, together with those from the literature, provide the basis of the following hypotheses for the present study: (i) that male ancestors who started smoking pre-puberty have grandchildren and/or great-grandchildren who have excess body fat; (ii) that any such associations are sex-specific, and (iii) will be found at both ages 17 and 24.

## Results

### Nomenclature for ancestors

**Figure 1:**
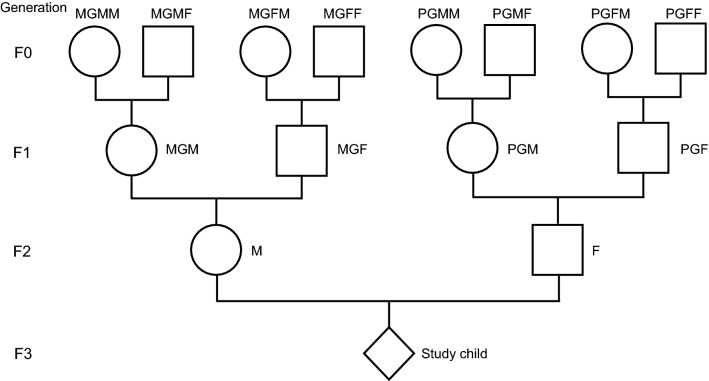
Family structure with nomenclature used.

The ways in which the ancestors are referred to in this paper are shown in Fig. 1. In brief, the four individuals on the maternal side of generation F0 are referred to as MGMM (maternal grandmother’s mother), MGMF (maternal grandmother’s father), MGFM (maternal grandfather’s mother) and MGFF (maternal grandfather’s father). The paternal side of generation F0 are labelled PGMM, PGMF, PGFM and PGFF with similar meanings. For the F1 generation, the labels are MGM and MGF on the maternal side and PGM and PGF on the paternal side. F2 is represented by M (mother) and F (father). F3 is the proband who is referred to as the great-grandchild, grandchild, or child depending on the generation whose onset of smoking is being considered (Fig. 1).

### Father’s smoking

Before considering associations between prepubertal onset of regular smoking in grandparents and great-grandparents we show the data originally analysed concerning the age at onset of smoking of the study fathers (F2s). Measurements of the fat and lean mass of the F3s at age 17 were available for 3645 ALSPAC probands (F3) for whom there was a record as to whether or not their fathers (F2) had ever smoked prior to the pregnancy. Of these, 49% of these fathers were, or had been, smokers and their progeny had a slightly greater mean fat mass than those whose fathers had never smoked (MD + 0.80 kg [95% CI + 0.13, + 1.74]; *P* = 0.010). Dividing the paternal smokers according to their age at starting regular smoking demonstrates the striking excess fat mass shown among the offspring of those who were regular smokers pre-puberty (< 11 years), with a drop in the excess fat mass as the age at onset of smoking increased (Table 1). The excess in fat mass for the 24-year-old offspring of men who had started smoking at age < 11 has been shown elsewhere to be increased upon adjustment (+ 11.22 kg [95% CI + 5.23, + 17.22]) with sons more affected than daughters^4^. In contrast, there were no associations of note between father’s age at onset of regular smoking and lean mass of his offspring at either 17 or 24 years of age.

**Table 1:** Unadjusted associations between mean fat mass of the F3 (probands) population according to age at which their fathers (F2) had started smoking regularly; only families where the study father had smoked are included.

Age (years) father	Offspring at age 17		Offspring at age 24	
Started smoking	n	MD [95% CI]	n	MD [95% CI]
		**Fat mass (kg)**		**Fat mass (kg)**
< 11	30	+ 8.06 [+ 4.18, + 11.9]	25	+ 9.73 [+ 5.40, + 14.1]
11–12	100	+ 1.63 [− 0.56, + 3.82]	86	+ 4.33 [+ 1.92, + 6.74]
13–15	523	+ 0.98 [− 0.13, + 2.09]	369	+ 0.84 [− 0.49, + 2.16]
16 +	1111	00 Reference	881	00 Reference
All known^b^	1764	*P* < 0.001^a^	1361	*P* < 0.0001^a^
		**Lean mass (kg)**		**Lean mass (kg)**
< 11	30	+ 0.63 [− 2.98, + 4.23]	25	+ 1.34 [− 2.56, + 5.25]
11–12	100	− 1.81 [− 3.85, + 0.22]	86	− 1.93 [− 4.11, + 0.24]
13–15	523	− 0.24 [− 1.27, + 0.79]	369	− 0.58 [− 1.77, + 0.62]
16 +	1111	00 Reference	881	00 Reference
All known^b^	1764	*P* = 0.293^a^	1361	*P* = 0.253^a^

CI, Confidence interval; MD, mean difference.

^a^*P* for trend.

^b^All smokers with known age at onset.

### Grandfather’s and great-grandfather’s onset of smoking prepuberty (< 13)

Data were available in sufficient numbers for MGF, PGF, MGMF and MGFF (Table 2). However, numbers who had started smoking prepuberty were small, and confidence intervals were wide (Tables 3, 4). Of the 24 associations calculated with fat mass, six (25%) showed similar significant associations at the two ages (PGF for all, and for girls only; MGFF for girls only). In contrast, our sensitivity analysis using lean mass showed only two (8%) of the associations were similar at the two ages (MGFF for girls only). Of the associations with both sexes combined, three of the fat mass associations were significant and none of the lean mass; three of the associations with fat mass showed significant interactions between the sexes, compared to none of the lean mass associations. Thus, there was evidence of more obvious associations with fat than lean mass.

**Table 2:** Numbers of ancestors known to have started smoking regularly before 17 years of age for whom there are F3 measures of fat and lean mass [numbers who had started smoking < 13 years of age are in square brackets].

Ancestor	All F3s	Male F3s	Female F3s
	**At 17**	**At 17**	**At 17**
MGF	975 [67]	415 [26]	560 [41]
PGF	437 [31]	177 [12]	260 [19]
MGMF	542 [20]	252 [9]	290 [11]
MGFF	415 [15]	179 [6]	236 [9]
	**At 24**	**At 24**	**At 24**
MGF	820 [50]	304 [16]	516 [34]
PGF	394 [25]	148 [7]	246 [18]
MGMF	471 [12]	194 [< 5]	277 [8]
MGFF	338 [16]	135 [7]	203 [9]

**Table 3:** Unadjusted associations between fat mass of the F3 population according to whether their grandfathers or great-grandfathers had started smoking regularly before the onset of puberty (< 13).

Smoked pre-puberty	All F3’s			F3 males			F3 females		
	n^a^	MD [95% CI]	*P*	n^a^	MD [95% CI]	*P*	n^a^	MD [95% CI]	*P*
**Fat mass at 17**				**Fat mass at 17**			**Fat mass at 17**		
MGF	67	0.48 [− 1.91, 2.87]	0.693	26	0.38 [− 3.25, 4.01	0.838	41	− 0.32 [− 3.10, 2.46]	0.821
PGF	31	**3.58 [0.20, 6.96]**	**0.019**	12	2.92 [− 1.87, 7.71]	0.231	19	**3.54 [− 0.51, 7.59]**	**0.043**
MGMF*	20	2.64 [− 1.74, 7.01]	0.237	9	**6.24 [0.23, 1.22]**	**0.021**	11	− 0.65 [− 5.97, 4.66]	0.809
MGFF*	15	2.99 [− 2.03, 8.01]	0.242	6	− 1.59 [− 8.37, 5.18]	0.642	9	**5.35 [− 1.01, 11.7]**	**0.050**
**Fat mass at 24**				**Fat mass at 24**			**Fat mass at 24**		
MGF	50	0.48 [− 2.35, 3.30]	0.740	16	− 1.37 [− 5.95, 3.22]	0.559	34	0.84 [− 2.67, 4.34]	0.639
PGF*	25	**3.10 [− 0.93, 7.14]**	**0.066**	7	− 4.16 [− 10.9, 2.54]	0.222	18	**5.49 [0.50, 10.5]**	**0.016**
MGMF	12	2.64 [− 2.91, 8.20]	0.350	< 5	0.26 [− 8.35, 8.86]	0.953	8	3.31 [− 3.69, 10.3]	0.352
MGFF	16	**4.17 [− 1.07, 9.41]**	**0.060**	7	2.15 [− 4.65, 8.95]	0.532	9	**6.10 [− 1.31, 13.5]**	**0.053**

Comparisons are with all ancestors who had started smoking between ages 13 and 16. *P* values in bold are using 1-tailed tests since the hypothesis was that there would be excess fat mass for early smokers.

MD, mean difference; CI, confidence interval; MGF, maternal grandfather; PGF, paternal grandfather; MGMF, maternal grandmother’s father; MGFF, maternal grandfather’s father.

^a^The number of grandchildren and great-grandchildren whose ancestor started smoking regularly < 13 years.

*Statistically significant interaction between the sexes.

**Table 4:** Unadjusted associations between fat mass of the F3 population according to whether their grandfathers or great-grandfathers had started smoking regularly before the onset of puberty (< 13), after eliminating the maternal or paternal grandfathers who had started smoking < 13.

Ancestor	All 17y grandchildren			17y grandsons			17y granddaughters		
	n	MD	*P*	n	MD	*P*	n	MD	*P*
**The grandfathers**									
MGF	63	4.38	0.482	24	0.896	0.644	39	− 0.11	0.941
PGF	29	**4.06**	**0.010**	11	**4.07**	**0.052**	18	3.43	**0.049**
**The great-grandfathers**									
MGMF [MGF < 13 excluded*	14	4.20	0.128	7	**9.79**	**0.003**	7	− 0.95	0.769
[PGF < 13 excluded*	20	2.54	**0.053**	9	**6.22**	**0.022**	11	− 0.82	0.761
MGFF < 13 [MGF < 13 excluded]	13	0.91	0.728	5	− 0.28	0.941	8	**6.59**	**0.022**
[PGF < 13 excluded	14	4.48	**0.045**	6	− 1.77	0.608	8	2.53	0.454
	**All 24y grandchildren**			**24y grandsons**			**24y granddaughters**		
**The grandfathers**									
MGF	48	0.64	0.661	15	0.92	0.704	33	0.79	0.659
PGF*	23	**3.12**	**0.071**	6	− 3.35	0.365	17	**4.86**	**0.030**
**The great-grandfathers**									
MGMF [MGF < 13 excluded	10	1.93	0.524	< 5	− 3.82	0.444	7	3.71	0.315
[PGF < 13excluded	12	2.63	0.350	< 5	0.17	0.969	8	3.32	0.348
MGFF [MGF < 13 excluded]	14	**4.75**	**0.047**	6	1.81	0.627	8	7.20	**0.034**
[PGF < 13 excluded	15	2.59	0.342	7	1.97	0.562	8	3.68	0.354

Comparisons are with ancestors who had started smoking between ages 13 and 16. *P* values in bold are using 1-tailed tests since the hypothesis was that there would be excess fat mass for early smokers.

Regarding the lean mass outcome, only one of the 24 associations was significant at *P* < 0.10: MGF smoking < 13 and grandsons at age 17: (mean difference 2.50 kg [95% CI + 0.13, + 4.87; *P* = 0.020; n = 41]) (Supplementary Table). This one association was considered no more than would be expected by chance, and the analyses of lean mass were not investigated further.

Of the two grandfathers, the PGF’s (but not the MGF’s) pre-pubertal onset of smoking showed an association with fat mass, with around 3 kg excess fat mass at both ages 17 and 24. There was some evidence that the granddaughters were affected more than the grandsons, with a significant interaction between the sexes at age 24 (Table 3).

**Figure 2:**
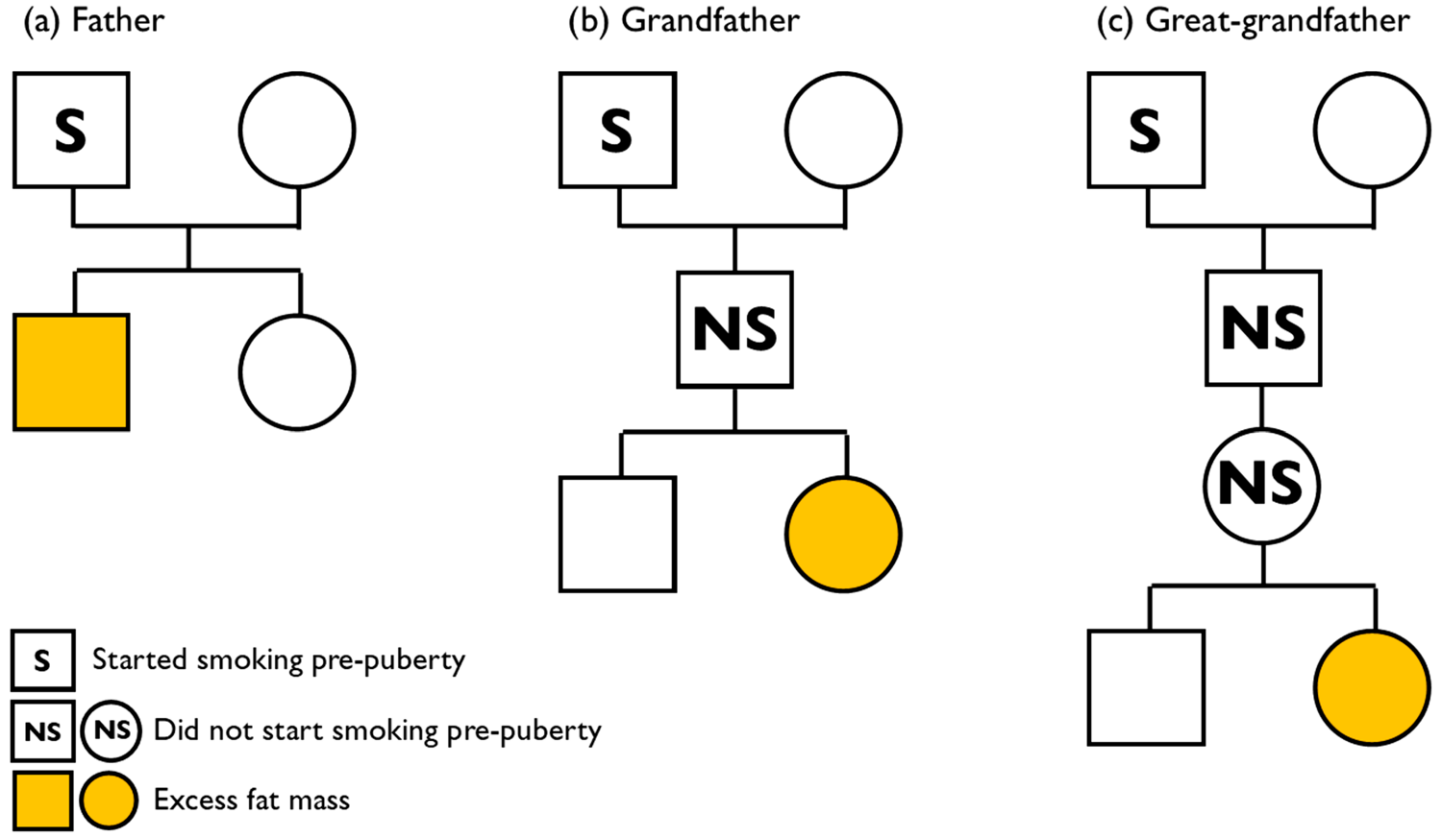
The way in which the onset of smoking pre-puberty is associated with extra fat mass in the subsequent family: (**a**) the father who starts smoking pre-puberty is associated with excess fat mass in his sons; (**b**) the paternal grandfather who starts smoking pre-puberty is associated with excess fat mass in his granddaughters; (**c**) the maternal grandfather’s father who starts smoking pre-puberty is associated with excess fat mass in his great-granddaughters.

The two great-grandfathers with sufficient numbers for analysis were the MGMF and MGFF. There was an association between the MGFFs who started smoking pre-puberty and excess fat (of 5.35 and 6.10 kg fat at ages 17 and 24 respectively) in their great-granddaughters but not great-grandsons (Table 3 and Fig. 2).

## Discussion

We set out to test whether exposure to the onset of regular smoking before puberty in ancestors had any detectable consequences on fat mass of the grandchildren and/or great-grandchildren. We used lean mass effects as a contrast, to ensure that any effect of fat mass was not true of another anthropometric measures. Based on the Ӧverkalix and the Berlin famine studies, and our earlier findings of an association between pre-pubertal onset of paternal smoking and increased fat mass in sons, but not daughters, we hypothesised that there would be sex-specific effects on grandchildren and great-grandchildren if their ancestor had begun to smoke regularly pre-puberty.

In order to be clear that the results were not due to associations with smoking in general, the analyses were confined to the grandfathers and great-grandfathers who had started smoking prior to 17 years of age. Thus, we compared the ancestors who had started smoking regularly pre-puberty (< 13 years) with those who started smoking later (13–16 years). We found that, despite small numbers and wide confidence intervals, there was evidence of increased fat mass in the granddaughters of the paternal grandfather and great-granddaughters of the maternal grandfather’s father at the two ages analysed (17 and 24 years).

Visualisation of the relationships with fat mass (Fig. 2) indicates that (a) the father who smoked pre-puberty was more likely to have a son (but not a daughter) with extra fat mass; (b) if the son of a man who had started smoking pre-puberty had a daughter, then she would be at increased risk of excess fat (although his sons would not); (c) if that granddaughter then reproduced, her daughters would, on average, have more body fat than those who started smoking later (although her sons would not). Unfortunately. We could not examine relationships associated with women (mothers, grandmothers, or great-grandmothers) smoking pre-puberty, as very few did so.

One explanation of our findings might be that the pre-puberty smokers had other features that might explain why their progeny had excess fat mass, such as a hereditary predisposition to obesity. However, there is substantial evidence that individuals who smoke regularly tend to have lower risk of obesity^10^, and ALSPAC data show that the fathers who had started smoking regularly before puberty had a lower BMI and waist circumference in adulthood than those who started smoking later in childhood (e.g. those who had started < 11 compared with those starting later had a reduced mean waist size of − 0.66 cm [95% confidence interval − 1.20, − 0.13; *P* = 0.004]). Consequently, if the F2 or F3 results had been able to adjust for the extra fat mass of their F0 or F1 ancestor, we would expect the effect sizes to have increased rather than decreased.

Our results provide some evidence of true transgenerational effects whereby an exposure to F0 prior to conception will not have a direct effect on grandchildren or great-grandchildren^11^. It is noteworthy that the associations indicated are related to obesity; it is generally recognised that obesity is a complex disorder caused by the interplay of genetics, epigenetics, and environmental factors^12^. It may be that the effects on the second (F2) and third (F3) generations after the initial prepubertal exposure might be the result of DNA methylation or other epigenetic markers being generated in consequence of the obesity of F1 and being inherited non-genetically by F2 and F3. However, before hypotheses are generated as to the mechanisms by which the effects we have shown may have occurred, it is important to seek confirmatory evidence from other studies.

This study has several advantages. First, it is based on a population selected by geography (area of residence) and not in regard to particulars of exposures or disorders. Second, we have shown that there are consistent results for measurements of F3 individuals at two ages, seven years apart; this is even though the populations of F3s studied at the two ages were not identical. Third, as we hypothesised, effects were sex-specific and therefore consistent with previous observations of ancestral exposures. Fourth, the different sex specific results from the different generations made a coherent pattern of inheritance.

The disadvantages are that the study parents (F2) were often not aware of the circumstances of the childhoods of their parents (F1) or grandparents (F0). Consequently, there was a large amount of missing data. Nevertheless, it is likely that most of the ancestors who did start smoking pre-puberty would have let that be known to their families—anecdotally it was something grandfathers and great-grandfathers boasted about, often with the claim that it had not done them any harm! Nevertheless, although the proportion of men born in the first part of the twentieth century had a rate of smoking cigarettes as high as 80–90%^13^, very few claimed to have started smoking before the age of 13. This resulted in very small numbers for analysis.

In conclusion, building on our previous demonstrations that when a study father had smoked regularly pre-puberty his sons were at increased risk of excess fat mass^1,8,9^, we have extended our studies to two preceding generations. We were able to show that associations with excess fat mass were also found when the paternal grandfather had smoked pre-puberty as well as when the maternal grandfather’s father had smoked pre-puberty. Numbers were small, however, and confidence intervals were wide. Consequently, the results should be treated with caution until substantiated in other studies.

The question arises as to whether these results, if substantiated, show a truly transgenerational effect. As described by King and Skinner^11^, preconception exposure-mediated epigenetic transgenerational inheritance can be induced by exposing the F0 generation to an environmental insult that can affect the epigenome of the germline. The germline, which eventually becomes the F1 generation, has been directly exposed to the environmental exposure, and is not considered to be transgenerational. Therefore, the F2 generation is considered to be the first nonexposed transgenerational offspring in this preconception exposure instance. Thus, phenotypic changes to the F2 and F3 generations after exposure of the male ancestors pre-puberty are considered transgenerational. We are not aware of previous human studies that have shown associations of pre-conception exposures in the F3 generation, and consider that this may be the first such finding.

## Material and methods

### The ALSPAC population

ALSPAC was designed to assess ways in which aspects of the environment and genes of individuals may interact to result in benefits and disadvantages to health and development^14^. It started during the pregnancies of women who were resident in a predefined area (that part of Avon that was within the South-West Regional Health Authority) and had an expected date of delivery between 1st April 1991 and 31st December 1992. Eligible women were contacted as early in pregnancy as feasible. They and their offspring were followed throughout pregnancy and then through childhood, adolescence and into adulthood. The collection of information is continuing. Data were collected using a variety of methods including questionnaires completed by the mothers, their partners and their offspring; analysis of biological samples; linkage to standard data sets, and hands-on examinations.

The initial number of pregnancies enrolled is 14,541 (for these at least one questionnaire has been returned or a “Children in Focus” clinic had been attended by 19/07/99). Of these initial pregnancies, there was a total of 14,676 fetuses, resulting in 14,062 live births and 13,988 children who were alive at 1 year of age. When the oldest children were approximately 7 years of age, an attempt was made to bolster the initial sample with eligible cases who had failed to join the study originally. As a result, when considering variables collected from the age of seven onwards (and potentially abstracted from obstetric notes) there are data available for more than the 14,541 pregnancies mentioned above. The number of new pregnancies not in the initial sample (known as Phase I enrolment) that are currently represented on the built files and reflecting enrolment status at the age of 24 is 913 (456, 262 and 195 recruited during Phases II, III and IV respectively), resulting in an additional 913 children being enrolled. The phases of enrolment are described in more detail in the cohort profile paper and its update^15–17^. The total sample size for analyses using any data collected after the age of seven is therefore 15,454 pregnancies, resulting in 15,589 fetuses. Of these 14,901 were alive at 1 year of age. Study data were collected and managed using REDCap electronic data capture tools hosted at the University of Bristol. REDCap (Research Electronic Data Capture) is a secure, web-based software platform designed to support data capture for research studies^18^. Please note that the study website contains details of all the data available through a fully searchable data dictionary and variable search tool: [https://www.bristol.ac.uk/alspac/researchers/our-data/]. Ethical approval for the study was obtained from the ALSPAC Ethics and Law Committee (ALEC; IRB00003312) and the Local Research Ethics Committees. Detailed information on the ways in which confidentiality of the cohort is maintained may be found on the study website: http://www.bristol.ac.uk/alspac/researchers/research-ethics/.

All methods were performed in accordance with the relevant guidelines and regulations. Informed consent for the use of data collected via questionnaires and clinics was obtained from participants following the recommendations of the ALSPAC Ethics and Law Committee at the time^19^.

As part of the original data collected during pregnancy, the questionnaires sent to the study mother and her partner (usually the father of the study child) included details of their childhood and adolescence, including the age at which they had started smoking regularly, together with other information on their smoking habits, and those of their parents (i.e., the study child’s grandparents). However, the smoking habits of these grandparents did not include details as to their ages when they had started smoking. Consequently, a recent endeavour has resulted in the sending of new questionnaires to those biological parents with whom the study was still in contact, to obtain further information on their parents and grandparents, including the age at which they had started smoking regularly. Questionnaires were mainly sent online, but for those who preferred paper alternatives, paper questionnaires were posted to them. Full details of the methodology and the questions asked can be found elsewhere^20^.

### The outcomes

Total fat and lean mass were estimated with the use of a Lunar Prodigy DXA scanner (GE Medical Systems Lunar, Madison, WI). The scans were visually inspected and realigned when necessary. For the present study we have used the measurements of fat and lean mass collected at face-to-face clinics at the ages of 17 (approximating to the end of puberty) and 24 years (early adulthood). The fat mass was used as our primary outcome hypothesis. Lean mass was used as a sensitivity analysis or comparison outcome to assess whether the associations with fat mass were distinct from those shown with lean mass.

### Statistical analyses

As we have indicated, earlier analyses using this cohort had shown associations between the fathers’ onset of regular smoking pre-puberty and increased fat mass in their offspring. Here we extend this analysis to demonstrate the associations between age at commencement of smoking and the outcomes to their grandchildren and great grandchildren. Given the findings with the fathers’ prepubertal onset of regular smoking, we examined possible associations with the pre-puberty onset of smoking of the two grandfathers. Because there is evidence that puberty started at later ages in the first half of the twentieth century^21^, we took < 13 years to denote the pre-puberty ages. Unfortunately, the numbers were too small for valid analysis for the paternal great-grandfathers PGFF and PGMF.

In general, we found extremely few grandmothers and great-grandmothers who had begun smoking prepuberty—numbers were too small for valid analysis. The numbers of reports of the age at onset of smoking of grandfathers and the maternal great-grandfathers pre-puberty were greater and were deemed sufficient for unadjusted analyses. The smoking information available only included whether the ancestor had started smoking in childhood (< 17) but did not include later onset. The analyses have therefore concentrated on comparing the families where the ancestor had started smoking aged < 13 (pre-puberty) with controls whose ancestors had started smoking in adolescence (13–16 inclusive); the ancestors of this generation who had either not smoked at all or who had started smoking after the age of 16 were omitted.

Because of the very small numbers in the index group, and lack of power, it was decided to consider 1-tailed *P* values of ≤ 0.10 since the study hypothesis concerned an increase (not a decrease) in fat mass. We had no prior hypotheses in regard to lean mass, so the tests were 2-tailed.

Initial analyses determined the unadjusted associations between each of the two grandfathers (F1s) and those of the two maternal great-grandfathers (F0s) where the numbers smoking pre-puberty were sufficient. The F3 outcomes were calculated separately using multiple regression analyses for (i) all children, (ii) males and (iii) females. In each instance the mean difference (MD) for both the fat and the lean mass of the target individuals (i.e., those whose ancestor started smoking pre-puberty) were compared with those who started smoking later in childhood using 95% confidence intervals. Presence of a significant interaction between the sexes was assumed when both the MD of the males was outside of the 95% confidence interval of the females and vice versa.

The numbers of individual male ancestors who had started to smoke in childhood at < 13 years and for whom data on age at starting to smoke regularly before age 17 were available altogether are shown in Table 1. The numbers smoking prepuberty were small, especially when stratified by sex. The grandparents had greater numbers for each sex, with maternal grandfathers having almost twice the totals of paternal grandfathers. Only two of the great-grandparents had sufficient numbers for analysis, both were on the maternal side. Consequently, the study was more likely to demonstrate evidence of association of prepubertal smoking in the maternal rather than the paternal line.

A further set of stratified analyses were carried out to determine whether associations shown with fat mass were the consequence of inherited likelihood of starting to smoke pre-puberty by omitting the great-grandchildren whose grandparents had started smoking pre-puberty. The lines of heredity shown in Fig. 2 show that, if our results are repeated, there is no possibility of a direct effect of a marker on the X chromosome of the paternal grandfather or great-grandfather, but that epigenetic markers on the autosomes are feasible.

## Supplementary Information

Supplementary Table S1.

## Data Availability

ALSPAC data is available to researchers for particular projects, provided no attempt is made to reveal the identities of the subjects. Guidelines for access are found on the ALSPAC website: www.bristol.ac.uk/alspac/researchers.
