# Supplementary material for: Human transgenerational observations of regular smoking before puberty on fat mass in grandchildren and great-grandchildren
Source: Sci Rep. 2022 Jan 21;12:1139. doi: 10.1038/s41598-021-04504-0 (PMC8782898; doi:10.1038/s41598-021-04504-0)
Supplement: Supplementary file 1 — Supplementary Table S1. [file 41598_2021_4504_MOESM1_ESM.pdf]

Human transgenerational observations of regular smoking before puberty on fat mass in grandchildren and great-grandchildren

Jean Golding*, Steven Gregory, Kate Northstone, Marcus Pembrey, Sarah Watkins, Yasmin Iles-Caven and Matthew Suderman

Bristol Medical School, Population Health Sciences, Bristol University, Bristol, UK

S1. Unadjusted associations between lean mass of the F3 population according to whether their grandfathers or great-grandfathers had started smoking regularly before the onset of puberty (<13). Comparisons are with all ancestors who started smoking aged 13-16. P values are using 2-tailed tests since the hypothesis was that there would be no association with lean mass.

**Smoked pre-puberty**	**All F3's**			**F3 males**			**F3 females**		
	n[a]	MD [95%CI]	P	n[a]	MD [95%CI]	P	n[a]	MD [95%CI]	P
	Lean mass at 17			*Lean mass at 17*			*Lean mass at 17*		
MGF	67	1.00 [-0.32, 2.31]	0.137	26	0.37 [-2.06, 2.80]	0.763	41	**2.50 [0.13, 4.87]**	**0.039**
PGF	31	-0.20 [-3.76,3.35]	0.911	12	1.01 [-2.74, 4.76]	0.596	19	0.03 [-1.88,1.93]	0.979
MGMF	20	-0.55 [-3.94,5.05]	0.808	9	2.68 [-1.55, 6.91]	0.831	11	-0.50 [-2.86, 1.86]	0.677
MGFF	15	0.67 [-4.51, 5.85]	0.799	6	0.88 [-4.20, 5.95]	0.733	9	2.19 [-0.71, 5.09]	0.138
	Lean mass at 24			*Lean mass at 24*			*Lean mass at 24*		
MGF	50	-.58 [-3.33, 2.17]	0.680	16	1.37 [-2.12, 4.86]	0.441	34	0.48 [-1.30, 2.26]	0.596
PGF	25	-.33 [-4.18, 3.51]	0.865	7	0.15 [-5.49, 5.79]	0.959	18	1.75 [-0.72, 4.23]	0.165
MGMF	12	0.24 [-1.02, 1.49]	0.712	<5	3.00 [-4.49, 10.5]	0.430	8	-0.63 [-3.39, 3.26]	0.970
MGFF	16	2.91 [-1.93, 7.74]	0.238	7	1.41 [-4.16, 6.98]	0.616	9	2.67 [-0.56, 5.89]	0.104

[a]The number of grandchildren and great-grandchildren whose ancestor started smoking regularly <13 years; *statistically significant interaction between the sexes; MD = mean difference in lean mass; CI = confidence interval; MGF = maternal grandfather; PGF = paternal grandfather; MGMF = maternal grandmother's father; MGFF = maternal grandfather's father.
